# Effect of serum IL-6, CRP, and MMP-9 levels on the efficacy of modified preperitoneal Kugel repair in patients with inguinal hernia

**DOI:** 10.1515/med-2024-1066

**Published:** 2025-04-23

**Authors:** Lifang Li, Renjie Cui, Wanli Ma, Kunhou Yao

**Affiliations:** Department of Gerneral Surgery, Huaihe Hospital of Henan University, Kaifeng, 475000, China; Department of Gerneral Surgery, Huaihe Hospital of Henan University, No. 115 Ximen Street, Gulou District,, Kaifeng, 475000, China

**Keywords:** modified Kugel repair, inguinal hernia, IL-6, CRP, MMP-9

## Abstract

**Objective:**

To evaluate the effect of serum IL-6, C-reactive protein (CRP), and matrix metalloproteinase-9 (MMP-9) levels on the efficacy of modified preperitoneal Kugel repair in patients with inguinal hernia.

**Methods:**

Clinical records of 42 patients with inguinal hernias who underwent modified preperitoneal Kugel repair were retrospectively analyzed. Serum IL-6, CRP, and MMP-9 were detected before surgery and after surgery. The patients were divided into the corresponding high-expression group and low-expression group. The basic data and clinical characteristics of patients were analyzed and compared, as well as postoperative indexes.

**Results:**

In patients with inguinal hernia, serum IL-6, CRP, and MMP-9 increase first and then decrease after surgery, reaching the peak value around 24 or 48 h after surgery. Patients with high-expression of IL-6, CRP, and MMP-9 had longer hospital stays and time to return to normal activities, and were more likely to have chronic abdominal pain. In addition, high-expression of IL-6 and CRP had a higher probability of postoperative VAS values and wound infection, and high-expression of IL-6 and MMP-9 were also more likely to have wound healing injuries.

**Conclusions:**

Serum IL-6, CRP, and MMP-9 levels in patients with inguinal hernia can affect the efficacy of modified preperitoneal Kugel repair and the prognosis of patients.

## 1 Introduction

More than 20 million abdominal mural hernias are repaired each year worldwide. The most common type of hernias, inguinal hernias are caused by the protrusion of visceral or adipose tissue in the inguinal or femoral ducts [1–3]. Due to the slow recovery and the recurrence rate of more than 10%, surgery is the best treatment, namely femoral groove hernia repair. However, most patients suffer from complications such as chronic abdominal pain and wound infection [4,5]. Therefore, the study of the factors affecting the curative effect of inguinal hernia surgery has a positive and critical role in improving the prognosis of patients.

In 1999, Kugel’s team introduced Kugel hernia repair for the first time, and performed 808 hernia repair operations within 54 months, among which only five recurrent cases and two cases of wound infection were performed, and the patients recovered well after surgery [6]. Modified Kugel herniorrhaphy is a frequently performed surgical procedure due to its prevalence as a common pathology [7,8]. Kugel repair is a preperitoneal inguinal hernia repair technique without a posterior approach through the inguinal canal. Modified Kugel repair can expose the surgical field more clearly and optimize the surgical process. The modified Kugel procedure achieved complete tension-free repair by placing a double elliptical patch in the preperitoneal space and adding a reinforced patch. Compared with the original Kugel surgery, modified Kugel repair has advantages such as minimally invasive, less suture, less postoperative pain, faster recovery, fewer complications, and low recurrence rate, as well as the advantages of traditional inguinal hernia repair through the anterior approach, short learning curve, and short operation time [9]. At present, modified Kugel repair is one of the representative surgical methods of open preperitoneal repair in clinical practice, which is widely applied. However, due to the implant of prosthesis materials during surgery, wound infection may be caused, resulting in wound healing injury and aggravating chronic abdominal pain and other sequelae [10,11]. In addition, the metabolic response of surgery leads to gluconeogenesis and increased protein synthesis in the acute phase [12], which is associated with metabolic and immunoactive cytokines such as tumor necrosis factor, interleukin-1 (IL-1), and IL-6 [13,14]. Matrix metalloproteinase-9 (MMP-9) plays a key role in wound healing after surgery, and the physical condition of patients before surgery has a great impact on the treatment and prognosis. Based on this, the study speculates that factors related to preoperative inflammation and wound healing may affect the prognosis of patients with inguinal hernia undergoing modified Kugel surgery.

Patients were first divided into the corresponding high-level group and low-level group by the median preoperative serum IL-6, C-reactive protein (CRP), and MMP-9. Subsequently, the patient’s medical records and follow-up records for up to 6 months were sorted out and analyzed, and the basic data, clinical characteristics, postoperative complications, length of stay, and time to return to normal activities were compared.

Therefore, there is a need to investigate the levels of these biomarkers and postoperative clinical outcomes in patients with modified Kugel repair. The aim of this study was to evaluate the preoperative clinical characteristics as well as postoperative recovery of patients with different biomarker levels in a patient cohort.

## 2 Materials and methods

### 2.1 Patients

Clinical records of 68 patients with inguinal hernia undergoing modified preperitoneal Kugel repair admitted to Huaihe Hospital of Henan University from December 2018 to July 2020 were retrospectively analyzed. Forty-two of the patients met the inclusion criteria and were subsequently examined based on patient records, including length of stay, degree of pain (Visual Analogue Scales [VAS]), inflammation (signs of redness, swelling, heat, and pain in the wound after surgery), wound healing, chronic abdominal pain, recurrence rate, and time to return to normal activities during a 3-month follow-up. Exclusion criteria were (1) patients with recurrent hernia or bilateral hernia, (2) pregnant patients, (3) patients with immune-related diseases, and (4) patients with malignant tumors.

### 2.2 Treatments

The surgery was performed by an experienced preperitoneal Kugel repair surgeon. All patients were operated on under local anesthesia and vital signs were monitored using a cardiac monitor. A modified Kugel hernia patch (Bard Davol, Needham, MA, USA) was placed as suggested [15]. In short, a 4–5 cm incision was made in the inguinal ligament. After determining the location of the hernia, the prepared patch was inserted into the preperitoneal space and fixed on the fascia transversalis and inguinal ligament with sutures. Finally, the inguinal canal and subcutaneous tissue were closed using absorbable sutures.

### 2.3 Observation indicators and detection methods

No anti-inflammatories were given before surgery, and anti-inflammatory drugs were given within 14 days after surgery. Meanwhile, 5 mL of intravenous blood samples was collected from patients before surgery and on Days 1, 2, 7, and 14 after surgery. After centrifugation at 3,000 rpm for 5 min, the sample was stored at −80°C. Serum IL-6, CRP, and MMP-9 levels were measured by ELISA kit (Thermo Fisher Technology).

The pain degree of postoperative patients was evaluated by VAS on Day 2 after surgery; 0 indicates no pain and 10 indicates the most severe pain. Patients with severe pain are treated with 100 mg of meperidine hydrochloride if needed.

Patients were categorized into corresponding high and low level groups based on the median preoperative serum IL-6, CRP, and MMP-9. Subsequently, the patients’ medical records and follow-up records up to 6 months were compiled and analyzed to compare the basic information, clinical characteristics, postoperative complications, hospitalization time, and time to return to normal activities.

### 2.4 Analysis of data

The sample size of the study was estimated using G*Power software version 3.1.9.2 with a significance level of *α* = 0.05, power of 1−*β* = 0.8, effect size of *d* = 0.7, and two-sided testing, requiring a minimum sample size of 40. Statistical analysis was conducted using SPSS 19.0 software (SPSS, Chicago, IL, USA). For continuous variables, Shapiro–Wilk and Levenes test were used to test normal distribution and homogeneity of variance. Data conforming to normal distribution shall be expressed as mean ± standard deviation, and those not conforming shall be expressed as median (25th and 75th percentiles). Use student *t* “test or modified *t*” test for data satisfying normal distribution, Mann–Whitney *U* test is used for non-normal distribution data, and Chi square test for discrete variables. *p* < 0.05 was considered statistically significant.


**Informed consent:** Written informed consent was provided by all patients prior to the study start.
**Ethical approval:** The present study was approved by the Ethics Committee of Huaihe Hospital of Henan University (No. 20180603HN-11). All procedures were performed in accordance with the ethical standards of the Institutional Review Board and the Declaration of Helsinki, and its later amendments or comparable ethical standards.

## 3 Results

### 3.1 Basic data and clinical features of patients

A retrospective analysis of 42 patients was performed. First, patients were divided into low- and high-expression groups of IL-6, CRP, and MMP9 by analyzing their median serum levels before surgery. Subsequently, the basic data and clinical characteristics were summarized. There were no statistical differences in age, gender, location, classification, incision length, and operative time between the low-IL-6 level group and the high-IL-6 level group (Table 1). Similarly, the basic data and clinical characteristics of patients with high and low level of CRP and MMP-9 were similar, with no statistical difference (Tables 2 and 3). Therefore, the relevant indicators after surgery are comparable.

**Table 1:** Demographic and clinical characteristics of patients with low or high IL-6 level

	IL-6 low-level (*n* = 21)	IL-6 high-level (*n* = 21)	*p*
Age (years)	60[54–65]	66[48–70]	0.284
BMI (kg/m^2^)	23.4[21.2, 24.6]	23.1[21.3, 24.5]	0.451
**Gender**			0.432
Male	16(76.2)	18(85.7)	
Female	5(23.8)	3(14.3)	
**Location of hernia**			0.757
Right	12(57.1)	11(52.4)	
Left	9(42.9)	10(47.6)	
**Nyhus classification**			0.846
Type 1: Indirect, small	8(38.1)	9(42.9)	
Type 2: Indirect, medium	5(23.8)	6(28.6)	
Type 3A: Direct	4(19.0)	4(19.0)	
Type 3B: Indirect, large	4(19.0)	2(9.5)	
Length of incision (cm)	4.4[4.2–4.6]	4.6[4.3–4.8]	0.211
Operative time (min)	53[48–60]	50[40–60]	0.528

Continuous variables were compared for differences between groups using Mann–Whitney *U* test, while discrete variables were compared using Pearson chi square.

**Table 2:** Demographic and clinical characteristics of patients with low or high CRP level

	CRP low-level (*n* = 21)	CRP high-level (*n* = 21)	*p*
Age (years)	62[55–68]	60[45–67]	0.513
BMI (kg/m^2^)	23.4[21.3, 24.5]	23.3[21.1, 24.6]	0.426
**Gender**			0.172
Male	17(81.0)	13(61.9)	
Female	4(19.0)	8(38.1)	
**Location of hernia**			0.929
Right	13(61.9)	10(47.6)	
Left	11(52.4)	8(38.1)	
**Nyhus classification**			0.772
Type 1: Indirect, small	7(33.3)	10(47.6)	
Type 2: Indirect, medium	6(28.6)	5(23.8)	
Type 3A: Direct	5(23.8)	3(14.3)	
Type 3B: Indirect, large	3(14.3)	3(14.3)	
Length of incision (cm)	4.4[4.1–4.6]	4.6[4.4–4.8]	0.052
Operative time (min)	58[49–60]	48[40–52]	0.091

Continuous variables were compared for differences between groups using Mann–Whitney *U* test, while discrete variables were compared using person chi square.

**Table 3:** Demographic and clinical characteristics of patients with low or high MMP9 level

	MMP9 low-level (*n* = 21)	MMP9 high-level (*n* = 21)	*p*
Age (years)	66[52–72]	60[54–65]	0.064
BMI (kg/m^2^)	23.3[21.3, 24.6]	23.2[21.2, 24.5]	0.475
**Gender**			0.432
Male	18(85.7)	16(76.2)	
Female	3(14.3)	5(23.8)	
**Location of hernia**			0.663
Right	10(47.6)	13(61.9)	
Left	7(33.3)	12(57.1)	
**Nyhus classification**			0.376
Type 1: Indirect, small	11(52.4)	6(28.6)	
Type 2: Indirect, medium	7(33.3)	4(19.0)	
Type 3A: Direct	3(14.3)	5(23.8)	
Type 3B: Indirect, large	2(9.5)	4(19.0)	
Length of incision (cm)	4.5[4.4–4.8]	4.5[4.1–4.6]	0.096
Operative time (min)	50[40–58]	56[48–62]	0.154

Continuous variables were compared for differences between groups using Mann–Whitney *U* test, while discrete variables were compared using Pearson chi-square.

### 3.2 Changes in serum IL-6, CRP, and MMP-9 levels in patients

**Figure 1:**
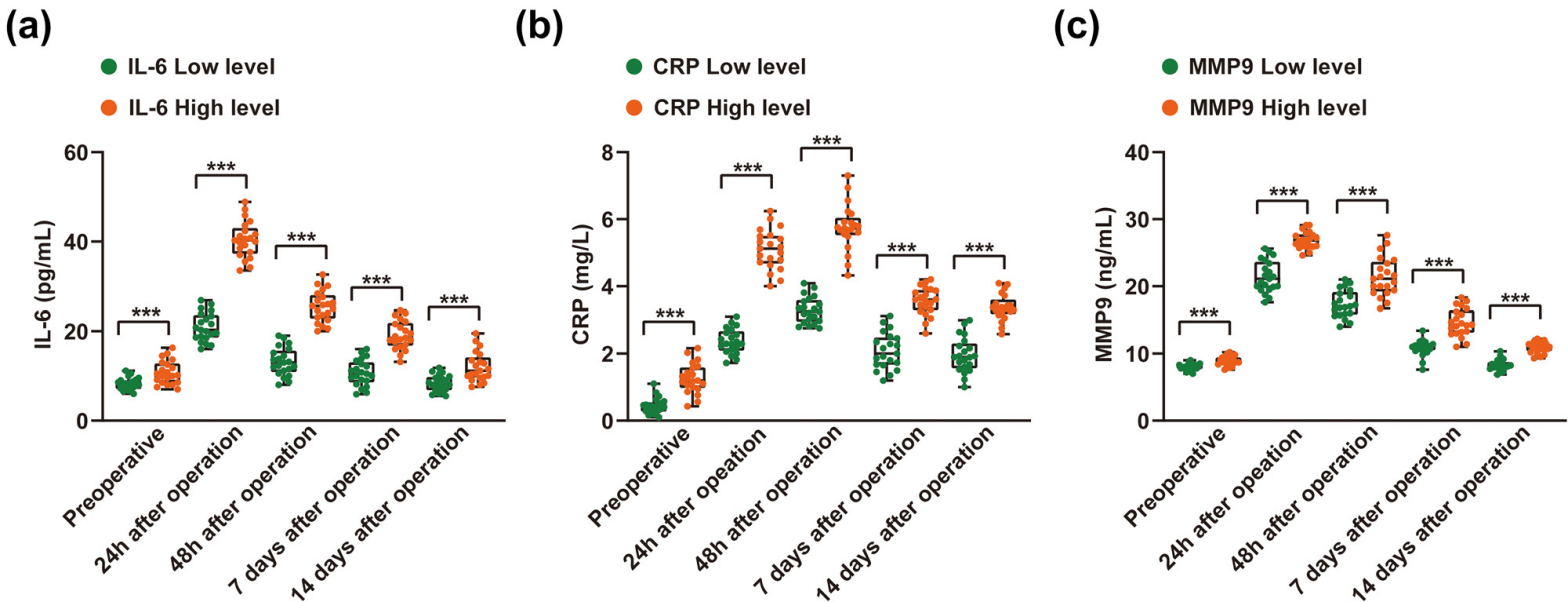
Changes of serum IL-6, CRP, and MMP-9 levels in patients. (a)–(c) Changes of serum IL-6, CRP, and MMP-9 level. Use student’s *t* ‘test or modified *t*’ test for data satisfying. ***, *p*＜0.001.

Preoperative and postoperative serum data were collected, and changes in IL-6, CRP, and MMP-9 were analyzed. IL-6 in all patients increased and then decreased after surgery, and the highest value appeared around 24 h, while the value was similar to that before surgery on Day 14. Meanwhile, patients with high IL-6 level also had higher serum IL-6 levels after surgery. In addition, serum CRP and MMP-9 levels also showed a trend of first increasing and then decreasing, and the serum CRP and MMP-9 levels in the high-level group were higher than those in the low-level group. However, CRP level was highest near 48 h after surgery, while MMP-9 level was near 24 h (Figure 1).

### 3.3 Postoperative indexes of patients

By analyzing postoperative indexes, it was noticed that patients with high IL-6 level had higher VAS values as well as longer hospital stay and the time required to return to normal activities than patients with low IL-6 level. Meanwhile, patients with high-level of IL-6 were more likely to have complications of impaired wound healing, wound infection, and chronic abdominal pain, but had no difference in the recurrence rate (Table 4). Similar results were seen in patients with high CRP and MMP-9 level (Tables 5 and 6). Therefore, serum IL-6, CRP, and MMP-9 in patients with inguinal hernia before surgery will affect the surgical efficacy and prognosis of patients.

**Table 4:** Postoperative indexes of patients with low and high level of IL-6

	IL-6 low-level (*n* = 21)	IL-6 high-level (*n* = 21)	*p*
VAS values	2.0[1.0–3.0]	4.0][2.0–5.0]	<0.001
Hospital stay (days)	1.4[1.2–1.8]	2.8[2.0–4.6]	<0.001
**Postoperative complications**			
Impaired skin wound healing	4(19.0)	11(52.4)	0.024
Wound inflammation	1(4.8)	16(76.2)	<0.001
Chronic abdominal pain	5(23.8)	18(85.7)	<0.001
Recurrence and others	0(0.0)	1(4.8)	0.311
Return to normal activities (days)	4.6[2.5–6.5]	8.0[5.3–9.5]	<0.001

Continuous variables were compared for differences between groups using Mann–Whitney *U* test, while discrete variables were compared using Pearson chi-square.

**Table 5:** Postoperative indexes of patients with low and high level of CRP

	CRP low-level (*n* = 21)	CRP high-level (*n* = 21)	*p*
VAS values	2.0[1.0–2.0]	4.0[3.0–5.0]	<0.001
Hospital stay (days)	1.2[1.1–1.4]	2.8[2.5–4.6]	<0.001
**Postoperative complications**			
Impaired skin wound healing	5(23.8)	10(47.6)	0.107
Wound inflammation	2(9.5)	15(71.4)	<0.001
Chronic abdominal pain	7(33.3)	16(76.2)	0.005
Recurrence and others	1(4.8)	0(0.0)	0.311
Return to normal activities (days)	5.0[4.5–6.7]	8.8[7.0–11.5]	0.016

Continuous variables were compared for differences between groups using Mann–Whitney *U* test, while discrete variables were compared using Pearson chi-square.

**Table 6:** Postoperative indexes of patients with low and high level of MMP9

	MMP9 low-level (*n* = 21)	MMP9 high-level (*n* = 21)	*p*
VAS values	2.0[1.0–2.0]	2.0[1.0–3.0]	0.092
Hospital stay (days)	1.4[1.2–2.5]	3.5[3.2–5.7]	0.026
**Postoperative complications**			
Impaired skin wound healing	2(9.5)	13(62.0)	<0.001
Wound inflammation	7(33.3)	10(47.6)	0.346
Chronic abdominal pain	8(38.1)	15(71.4)	0.030
Recurrence and others	0(0.0)	1(4.8)	0.312
Return to normal activities (days)	5.2[4.3–6.5]	7.6[4.6–9.5]	0.033

Continuous variables were compared for differences between groups using Mann–Whitney *U* test, while discrete variables were compared using Pearson chi-square.

## 4 Discussion

Inguinal hernia is a common surgical condition caused by a defect or weakness of the deep transverse fascia in the inguinal region. At present, modified Kugel repair is one of the most widely used operations. However, most patients still have complications such as wound infection and chronic pain after surgery, resulting in certain restrictions on their life and work [16]. However, postoperative pain is affected by various factors, such as preoperative pain, intraoperative nerve damage, and inflammation associated with reticular materials [10,17]. Based on previous studies, the article reviewed the medical records of 42 patients and confirmed that preoperative serum levels of IL-6, CRP, and MMP-9 affected the prognosis of patients with inguinal hernia and were positively correlated with postoperative wound infection, but negatively correlated with postoperative pain, wound healing, length of stay, and time to return to normal activities.

Most surgical injuries are caused by the body’s inflammatory response. The modified Kugel repair is to place the patch in the preperitoneal space, completely cover the entire inguinal area, and enhance the repair of the weak inguinal area. The selected biological patch has a soft texture and a good fit with human tissue, and the patient’s discomfort after implantation is relatively light [18]. However, some current studies have confirmed that after implantation of the prosthesis material, the body will begin to experience acute inflammation and subsequent chronic fibroblast changes resulting in tissue scarring and the formation of fibrosis or granuloma. In some cases, the inflammatory process will also cause damage to the surrounding tissues and lead to chronic pain in patients [19], and studies have found that IL-6 plays an important role in inducing and maintaining pain [20]. This inflammatory response appears to be characterized by elevated IL-6 and CRP levels [21–23]. Our results were similar, with increased serum IL-6 and CRP levels after surgery. IL-6 mainly regulates the acute phase response and induces the synthesis of various plasma proteins in hepatocytes, leading to the synthetic expression of acute phase proteins such as CRP 22. In this study, IL-6 reached its peak elevation 24 h after surgery, but CRP reached its peak about 48 h after surgery. This is consistent with the known role of IL-6 in inducing CRP synthesis. In addition, IL-6 reached its peak 24 h after surgery. We speculate that postoperative weakness may be a potential cause of pro-inflammatory effects in patients.

CRPs are pro-inflammatory factors that can be used to assess the severity of biological trauma based on cytokine secretion. IL-6 is a class of factors with bidirectional effects, and its anti-inflammatory activity is mediated by classical signals, while the pro-inflammatory response is mediated by *trans* signals [24]. Importantly, IL-6 is a sensitive marker of tissue damage and is involved in regulating the host defense mechanism [25], while CRP, an acute phase protein, is closely related to the severity of inflammation and prognosis of patients. Patients with high IL-6 or CRP level had less postoperative pain, a shorter hospital stay, and a shorter time to return to daily activities. Patients’ physical conditions before surgery, such as inflammation, will significantly increase the incidence of complications such as wound infection, which is unfavorable to body recovery. In this study, our results indicate that patients with high IL-6 or CRP level are more likely to have chronic abdominal pain, wound infection, and wound healing injury than those with low-level. This indicates that preoperative serum IL-6 and CRP levels affect the prognosis of patients with inguinal hernia.

In addition, the prosthesis materials can induce an acute inflammatory response, followed by tissue fibrosis and granuloma formation, and the patient develops chronic foreign body reactions [26]. Extracellular matrix molecules are critical in tissue repair, and MMPs are involved in the degradation of extracellular matrix. Increased level of MMP-9 is related to the decreased amount of collagen and tissue inflammatory response, which affects wound healing [27]. Our results showed that serum MMP-9 level in patients increased to a peak about 24 h after surgery and then decreased. In addition, serum MMP-9 levels were always higher in patients with high MMP-9 level compared to those with low-level. Meanwhile, patients in the high-level group had a longer hospital stay and return to normal activities, and had a higher probability of wound healing injury. This may be because increased MMP-9 levels lead to a delayed wound-healing response and less collagen deposition [28]. Therefore, patients’ preoperative serum MMP-9 levels may also influence the efficacy of modified Kugel hernia repair after treatment.

## 5 Limitations

However, the sample size was small and 6-month follow-up may not be sufficient to assess the correlation between preoperative serum and prognosis of patients with modified Kugel repair. Second, although the trial was subject to strict quality control, it could not be treated completely objectively because it was a single-center study. In addition, we will increase the frequency of detection of serum factors before and after surgery in subsequent studies, and use more accurate data for analysis.

## 6 Conclusion

Preoperative serum IL-6, CRP, and MMP-9 affect the efficacy of modified Kugel repair, and the higher the preoperative serum IL-6, CRP, and MMP-9 levels, the more likely patients were to have postoperative pain, wound infection, wound healing injury, and chronic abdominal pain as well as longer hospital stays and time to normal activities.
